# Machine learning and bioinformatics approaches for classification and clinical detection of bevacizumab responsive glioblastoma subtypes based on miRNA expression

**DOI:** 10.1038/s41598-022-12566-x

**Published:** 2022-05-23

**Authors:** Jian Shi

**Affiliations:** Department of Neurology, Department of Veterans Affairs Medical Center, University of California, San Francisco, CA 94121 USA

**Keywords:** Cancer, Biomarkers

## Abstract

For the precise treatment of patients with glioblastoma multiforme (GBM), we classified and detected bevacizumab (BVZ)-responsive subtypes of GBM and found their differential expression (DE) of miRNAs and mRNAs, clinical characteristics, and related functional pathways. Based on miR-21 and miR-10b expression z-scores, approximately 30% of GBM patients were classified as having the GBM BVZ-responsive subtype. For this subtype, GBM patients had a significantly shorter survival time than other GBM patients (*p* = 0.014), and vascular endothelial growth factor A (VEGF) methylation was significantly lower than that in other GBM patients (*p* = 0.005). It also revealed 14 DE miRNAs and 7 DE mRNAs and revealed functional characteristics between GBM BVZ subgroups. After comparing several machine learning algorithms, the construction and cross-validation of the SVM classifier were performed. For clinical use, miR-197 was optimized and added to the miRNA panel for better classification. Afterwards, we validated the classifier with several GBM datasets and discovered some key related issues. According to this study, GBM BVZ subtypes can be classified and detected by a combination of SVM classifiers and miRNA panels in existing tissue GBM datasets. With certain modifications, the classifier may be used for the classification and detection of GBM BVZ subtypes for future clinical use.

## Introduction

Glioblastoma multiforme (GBM) is the most common and the most lethal malignant brain tumor. Despite decades of intensive efforts to optimize the treatment of glioblastoma, the outcomes of GBM patients are still disappointing, with a median life expectancy of ~ 15 months after diagnosis^1^. The main reason for this poor outcome is that despite extensive investigations of the pathogenesis of GBM, the various genetic risk factors involved in the development of the disease remain poorly understood. However, because of substantial large-scale genome sequencing studies, knowledge of GBM epigenetics and genetics has been growing very fast and going very far. Facing the vast genetic information of brain cancer, we have to work harder to understand the true meaning of genetics under the surface, and to more accurately classify them through those informations rather than just vague slides^2^. To date, early detection and accurate classification of tumors is still the most effective method to achieve the best efficacy for patients.

MicroRNAs (miRNAs) are small noncoding RNAs that are approximately 22 nucleotides long, but they can bind to messenger RNAs (mRNAs) and mainly play an inhibitory role in gene expression in a posttranscriptional manner^3–5^. Previous studies have shown that changes in miRNA expression in tumor tissues and body fluids are different from those in normal tissues or different GBM subtypes^6^. These changes involve various aspects of GBM, including tumor initiation, aggressiveness, responses to drug treatments, and patient survival rates. Profiling and studying these miRNA expression differences can help to further classify GBM^7–10^. For example, based on miRNA and mRNA expression, five clinically and genetically distinct glioblastoma subclasses were classified, including oligoneural, radial glial, neural, neuromesenchymal, and astrocytic precursor glioblastoma^7^. However, all these glioma subtypes classified with multiple miRNAs are difficult to accurately detect by traditional biomarkers and thresholds. In addition, there are fewer classifications related to drug responses, especially for bevacizumab (BVZ) treatment.

Treatments for GBM include resection, radiation, chemotherapy, immunotherapy, and recent anti-angiogenic therapy, such as BVZ treatment, which targets vascular endothelial growth factor A (VEGF). Anti-angiogenic treatment can significantly reduce the early stage of contrast enhancement during imaging studies and is therefore considered to have a high radiological response rate^11,12^. However, BVZ treatment did not prolong the overall survival of GBM patients with these radiological responses. Despite this, many physicians still believe that certain patients seem to benefit significantly from bevacizumab treatment, so more research should have been conducted to better identify this patient subgroup^13^. Researchers also want to know what happens to these patients after receiving BVZ treatment, and what we can do for them in the near future.

For early detection and accurate classification of cancers, several machine learning algorithms have been applied to microarray datasets, including support vector machines (SVMs), random forest (RF)^14^, and neural network (NN)^15^. However, previous studies have shown that among popular techniques for multicategory classification of gene expression profiling datasets, SVMs have a dominant role, significantly outperforming all other methods, especially for small datasets and binary classification^16,17^. SVMs were first introduced by Vapnik^18^. Recently, it has shown effectiveness in many pattern recognition problems^19^, such as cancer histopathology image analysis and recognition, and they usually provide better classification performances than many other classification techniques. To identify GBM patients from normal patients, Teplyuk et al. reported using an SVM method based on miRNA expression levels in CSF to separate them^20^. To date, there have been no successful reports using this method in combination with biomarkers to classify and detect cancer subtypes. To achieve better results using SVM methods in GBM classification, prediction, and detection, it is also very important to collect a certain number of datasets and select a suitable kernel function for SVMs. The heterogeneity of GBM complicates the classification of their responses to different treatments, especially considering races, different mutants and gene expression, and the clinical techniques used to obtain experimental datasets.

In this study, we defined and classified GBM BVZ-responsive subtypes based on the expression of the miRNA panel from existing clinical studies and transcription profiling. Then, to verify this new GBM subtype, after using various bioinformatics methods, differential expression (DE) of miRNAs and mRNAs was found, and their related clinical characteristics, pathways, and functions between the subtypes were analyzed. Finally, several machine learning algorithms were constructed and compared based on the miRNA expression z-scores and clinical datasets, and the SVM classifier was selected for this study. To further use the classifier, the panel of miRNAs was optimized, and the classifier was modified. After certain modification and validation, this study may be used for GBM clinical sample classification, detection, and further clinical and basic research.

## Materials and methods

### Experimental procedure

**Figure 1:**
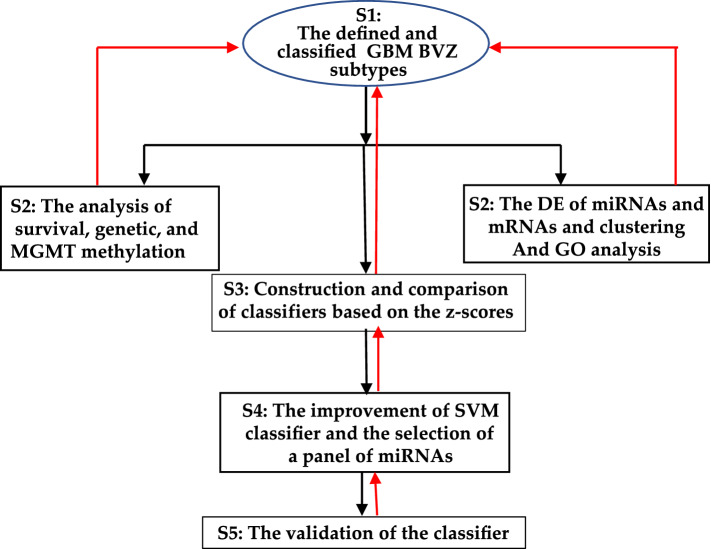
The experimental procedures. (**A**) The defined and classified BVZ subtypes of GBM. (**B**) This step includes several parts of the analyses. (**C**) The construction and comparison of classifiers based on the miRNA expression z-scores and clinical datasets. (**D**) The optimization of a panel of miRNAs, including miR-21, miR-10b, and miR-197, and the modification and cross-validation of the SVM classifier based on clinical datasets. (**E**) The validation of the classifier using other datasets. In this figure, black lines and arrows indicate the workflow, and red lines and arrows indicate that the results obtained call back and support the previous work or definition.

The experimental procedure included the following steps, as shown in Fig. 1. The first step was to define and classify the GBM BVZ-responsive subtype (abbreviated as the GBM BVZ subtype). According to the following criteria, all patients were divided into the GBM BVZ subtype and its control subgroup (BVZ nonresponsive) by using the Venn diagram method^21^. In the 2nd step, the GBM BVZ subtype was assessed, demonstrated, and compared to its control subtype through multiple bioinformatic and clinical analyses, including Kaplan–Meier survival curves, miRNA and mRNA clustering, clinical characteristics, and genetic and functional analyses. In the 3rd step, based on the expression z-scores of miR-21 and miR-10b in GBM BVZ subtypes, several machine learning algorithms, such as SVM, RF, and NN, were constructed and compared, and the SVM classifier was selected, in which the radial basisi function (RBF) was used as the kernel function. The 4th step was to prepare the SVM classifier for clinical use, find the best combination of miRNAs, perform cross-validation, and modify the classifier. Finally, the SVM classifier was examined and validated by using several datasets. In Fig. 1, black lines represent the workflow, and red lines represent the feedback support of the results.

### Experimental datasets

In the Cancer Genome Atlas (TCGA) pilot study, mRNA and miRNA expression profiles of GBM were generated using Affymetrix U133A, Affymetrix Exon 1.0 ST, custom Agilent 244 K, and Agilent miRNA array platforms. Several mRNA expression profiles were integrated into a single estimate of relative gene expression for each gene in each sample^22^. From TCGA portal (http://cancergenome.nih.gov/dataportal/), the expression z-scores of miRNA and mRNA and miRNA expression profiling were downloaded, especially including data_expression_merged_median_Zscore and data_expression_miRNA, and we also downloaded additional datasets, including clinicopathological annotations and methylation for glioblastoma patients.

### Software and statistical methods

In this study, the software and online tools used included MATLAB (R2021b and R2022a, MathWorks Inc., Natick, MA), R-project (version 4.0.4, www.r-project.org), MeV (version 4.9.0, MEV, LLC., Walnut Creek, CA, http://www.tm4.org/mev.html), the Venny diagram^21^, Gene Ontology (http://geneontology.org/), g:Profier (ELIXIR, Tartu, Estonia, https://biit.cs.ut.ee/gprofiler/gost), and SigmaPlot 14 (Systat Software, San Jose, CA).

In this study, the statistical methods included Student’s t-test, the standard Bonferroni adjusted t-test, and the Wilcoxon-Mann–Whitney test if the data distribution was not the standard distribution.

### Definition and classification of GBM BVZ subtypes

In this study, we defined the BVZ-responsive GBM subtype (abbreviated as the GBM BVZ subtype) as patients with high expression of miR-21 and miR-10b in their tumors. The GBM BVZ subtype was defined because high levels of miR-10b and miR-21 were observed in the serum, cerebrospinal fluid (CSF), and tumor tissues of some glioblastoma patients compared with normal control patients^20,23,24^. Most importantly, the expression levels of miR-10b and miR-21 were negatively correlated and significantly associated with decreased tumor diameters in the BVZ treated group, but not in the temozolomide (TMZ)-treated group.

High levels of miR-10b or miR-21 were defined as z-scores greater than zero, and low levels of miR-10b or miR-21 were defined as z-scores below or zero. Patients were selected and subdivided into four groups according to the expression z-scores of miR-10b and miR-21 using the R program. Then, using the Venn diagram^21^, the patient IDs of the GBM BVZ subtype with high expression of both miR-21 and miR-10b were obtained, while the patient IDs of other groups were also obtained, including the group with both miR-21 and miR-10b downregulated group (DD), miR-21 upregulated and miR-10b downregulated group (UD), and miR-21 downregulated and miR-10b upregulated group (DU). All three subgroups were combined as either the GBM BVZ nonresponsive subtype (abbreviated as the CON subtype) or the GBM control subtype.

### Hierarchical clustering of miRNAs and mRNAs of GBM BVZ subtypes

Based on patient IDs of GBM BVZ subtypes (BVZ and CON), the z-score matrices of miRNAs and mRNAs were obtained using the R program, and patients’ GBM BVZ subtypes were also labelled. Then, clustering and heatmaps of miRNAs and mRNAs were obtained by using MeV program^25,26^. Since two subgroups were given before clustering, this hierarchical clustering belongs to semi-unsupervised learning.

### Cell culture and GBM tissues

Four cancer cell lines were cultured in this study. All cell lines were purchased from the American Type Culture Collection (ATCC). All experiments followed the Biological Use Authorization (BUA) of VA Medical Center at San Francisco, California. The human U87 GBM cell line was cultured in 10% FBS in DMEM (Invitrogen, USA). Medulloblastoma (MB) cells (CHLA-01-MED) were cultured in DMEM: F12 medium (ATCC 30-2006) with 20 ng/mL human recombinant EGF, 20 ng/mL human recombinant basic FGF, and B-27. The PC3 prostate cancer cell line was cultured in 10% FBS in F-12 (Invitrogen, USA), and the DU145 prostate cancer cell line was cultured in 10% FBS in DMEM (Invitrogen, USA). Other details can be obtained from our previous study^27^.

We submitted used GBM samples to the study following an agreement approved by the Review Board of the University of California, San Francisco (UCSF). Three fresh human glioblastoma multiform specimens were collected from patients, including GBM10302 (age 49, female), GBM 9746 (age 70, male), and GBM10099 (age 55, male). Informed consent was obtained from all the participants included in the study. All tissue samples were obtained during the initial resection, and none of the patients had received prior chemotherapy or radiation therapy. The samples were immediately cut into pieces estimated at 30–40 mg per piece and stored in a − 80 °C freezer until use.

### Ethics approval and consent to participate

Informed consent was obtained from all three human samples. The ethics approval number of the Local of Ethics Committee of University of California is 10-01318, which is used to de-identify human biospecimens. These studies were in accordance with the ethical standards of the institutional research committee and with the 1964 Helsinki Declaration and its later amendments.

### Quantitative real-time PCR

Total RNA from all experimental cells and tissues was isolated by using a mirVana™ miRNA Isolation Kit (Thermo Fisher, USA), and the RNA quality and quantity were evaluated using a NanoDrop 2000 (Thermo Scientific, Waltham, MA). Reverse transcription (RT) of the RNAs was performed by using a first-strand cDNA synthesis system (Invitrogen, USA), according to the manufacturer’s protocol. Other details can be obtained from our previous research^27,28^.

Real-time PCR amplification was performed using a real-time PCR kit (TaKaRa, USA) following the manufacturer’s protocol. For all experiments, PC3 and DU145 cells were used as negative and technical controls to keep the machine settings, reagents and techniques at the same level for each experiment. The test samples were subjected to 40 cycles of PCR amplification. The experiments were performed using the QuantStudio 7 and 7900HT Fast Real-time PCR system (Applied Biosystems). Other details can be obtained from previous studies^27,28^. All experiments were repeated in duplicate for all samples and all miRNAs.

## Results

### GBM sample cohort

In this GBM sample cohort, there were 564 samples (544 untreated and 20 treated samples from 220 female and 344 male patients)^22^. In this study, we analyzed 409 GBM samples containing both mRNA and miRNA profiles and z-scores for each sample from untreated patients. All patients had been diagnosed with glioblastoma multiforme, some of which had more pathological information, including survival time, heredity and mutations, and methylation.

### Classification and clustering of miRNAs and mRNAs for GBM BVZ subtypes

**Figure 2:**
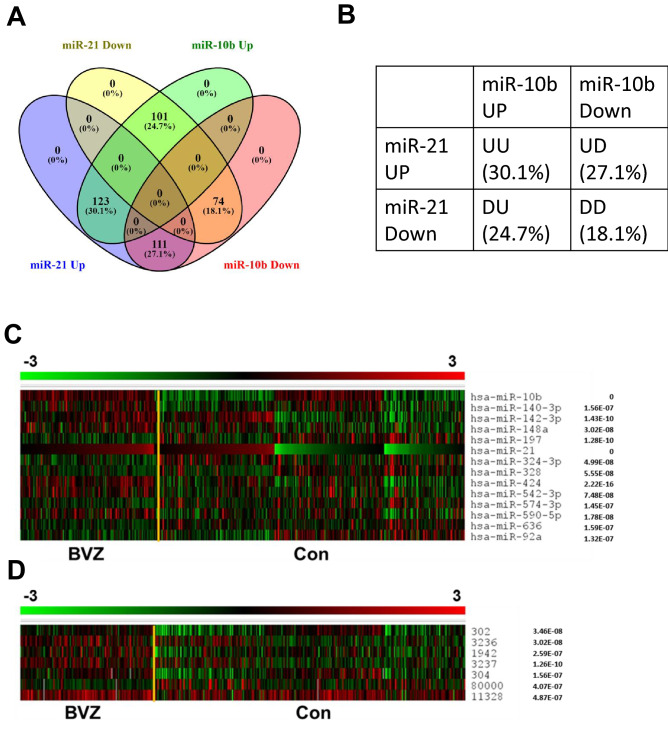
GBM BVZ subtypes and hierarchical clustering heatmaps of miRNAs and mRNAs. (**A**) The upregulated expression z-scores of miR-21 and miR-10b (UU) define the GBM BVZ subtype, which is present in ~ 30% of all GBM patients. (**B**) The table shows all four subgroups of GBM, their labels, and percentages. (**C**) Hierarchical clustering heatmap of miRNAs in GBM BVZ and CON subtypes. The DE 14 miRNAs and their *p*-values are listed on the right of the map, *p* = < 0.0001 vs the control, n = 409. (**D**) Hierarchical clustering heatmap of mRNAs in these two GBM subgroups. Those seven mRNA IDs and their *p*-values are listed on the right of the map, *p* = < 0.01 vs the control, n = 409. Rulers of miRNA and mRNA expression are above the pictures, where − 3 to 3 correspond to green to red. The yellow lines separate the GBM BVZ and its control subgroups, and the left sides of the lines are the GBM BVZ subtypes. If a *p*-value is less than 1.0E-10, the software will give zero as the *p*-value.

Among all GBM patients, the percentage of patients with the GBM BVZ subtype is very important for follow-up studies. Based on the defined criteria and using the Venn diagram tool^21^, the distribution of all glioblastoma patients was grouped into four subgroups, as shown in Fig. 2A and B. A total of 123 patients were classified as the GBM BVZ-responsive subtype, representing 30.1% of all GBM patients, who were highly responsive to BVZ treatment. Most importantly, a previous study showed that 33% of GBM patients responded to BVZ treatment compared to normal controls^29^. The other 286 patients, classified as the BVZ nonresponsive subtype of GBM, did not highly respond to BVZ treatment. According to the biological definition, we focused on these two GBM subtypes in the following studies. In addition, 286 control GBM patients were combined with three other subgroups, DD, UD, and DU, as shown in Fig. 2B.

Some DE miRNAs and mRNAs were found after the comparisons between GBM BVZ subtypes (BVZ and CON subtypes). After patient selection, DE miRNA analysis was performed by performing a t-test on the obtained z-score matrix to determine which miRNAs differed most between the subtypes. Following data statistic analysis, miRNAs were ranked by *p*-value with a cutoff at 0.0001 and standard Bonferroni correction for each subgroup. Specifically, 14 miRNAs between GBM BVZ subgroups passed the threshold as highly relevant miRNAs, including miR-10b, − 140-3p, − 142-3p, − 148a, − 197, − 21, − 324-3p, − 328, − 424, − 542-3p, − 574-3p, − 590-5p, − 636, and − 92a. Hierarchical clustering and DE miRNAs between subgroups were then visualized as a heatmap using MeV software, as shown in Fig. 2C. In this heatmap, these highly relevant miRNAs and their *p*-values are listed on the right, the GBM BVZ subgroup is on the left of the yellow line, and the control subgroup is on the right.

For mRNA DE analysis, clusters between these GBM BVZ subgroups were examined. Based on the matrix of these GBM BVZ subtypes classified above, significant genes were picked using Student’s t-test at *p* = 0.01 and FDC (false discovery correction, standard Bonferroni correction). Specifically, seven genes were found to pass the statistical threshold between these two subgroups as gene signatures of GBM BVZ subtypes. These seven gene IDs are 302 (ANXA2), 3236 (HOXD10), 1942 (EFNA1), 3237 (HOXD11), 304 (ANXA2P2), 80000 (GREB1L), and 11328 (FKBP9). DE gene expression between these two subgroups was represented as a heatmap using MeV software, as shown in Fig. 2D. In this map, the significant gene IDs and their p-values are listed on the right side of the map. These DE expression levels of miRNAs and mRNAs may suggest that the expression changes of miR-21 and miR-10b in glioblastoma have an important role in classifying glioblastoma patients into distinct subgroups.

### Clinical characteristics of GBM BVZ subtypes

**Figure 3:**
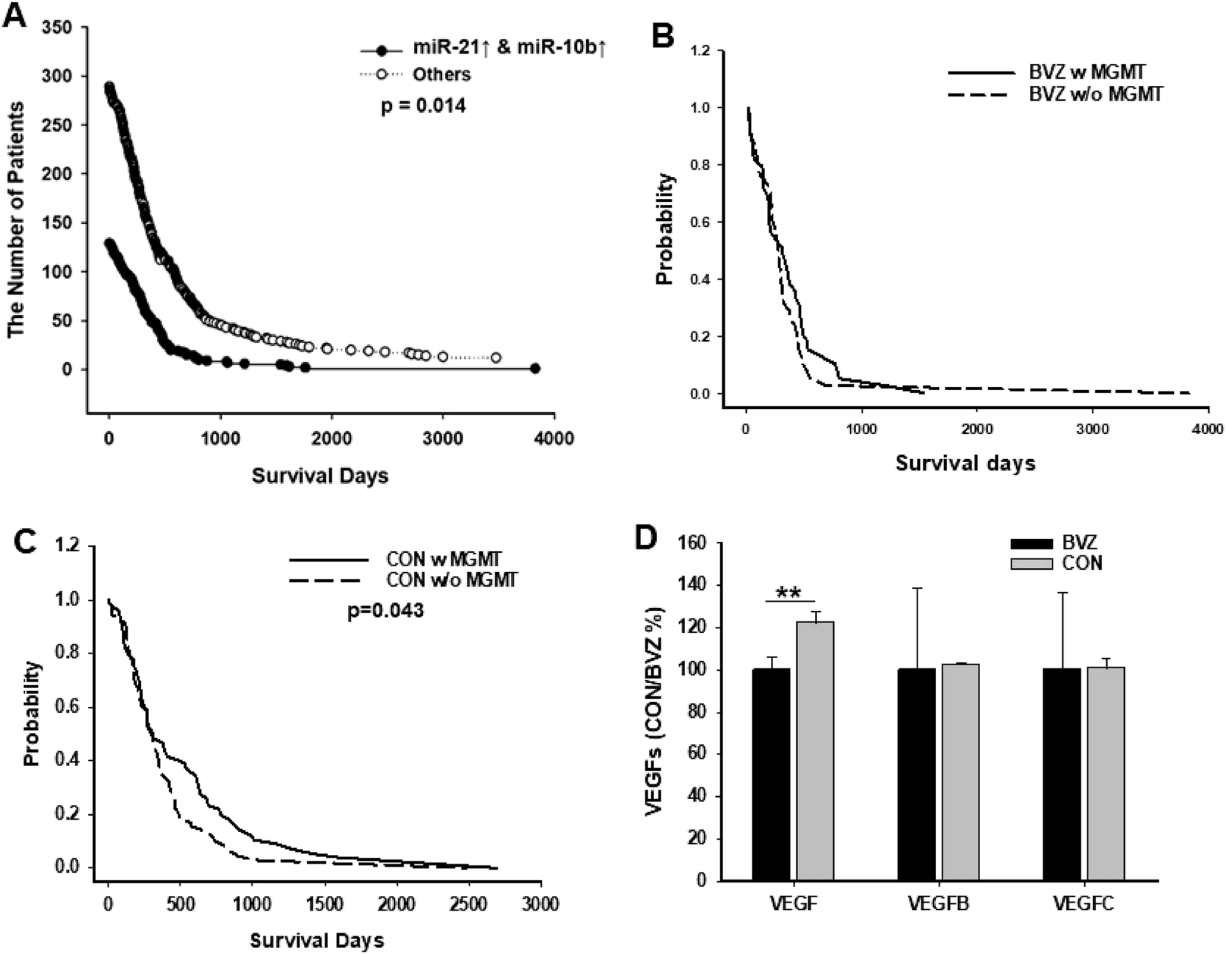
Distinct clinical characteristics of GBM BVZ subtypes. (**A**) Kaplan–Meier survival curves for the GBM BVZ subtype and the control (*p* = 0.014, Wilcoxon-Mann). (**B**) Kaplan–Meier survival curves for GBM BVZ patients in each subclass (w or w/o MGMT methylation) of the entire subtype are shown (*p* > 0.05). (**C**) Kaplan–Meier survival curves for GBM CON patients in each subclass are shown (*p* = 0.043, Wilcoxon-Mann). Black or dashed lines represent the survival of patients with or without MGMT methylation, respectively. (**D**) Comparisons of the gene methylation of VEGF, VEGFB, and VEGFC between GBM BVZ subtypes are shown. There was only one significant difference between GBM BVZ and CON subtypes in VEGF methylation (*p* = 0.005, Wilcoxon-Mann).

Differences in survival time between the two classified GBM BVZ subtypes suggested and supported the existence of a new BVZ subtype of GBM. GBM BVZ subtypes classified according to the high expression of miR-21 and miR-10b were absent in any of the subclasses based on miRNA consensus clustering^7,24,30^; thus, the GBM BVZ-responsive subtype is a new subtype of GBM. Based on this classification of glioblastoma, patients’ survival days were assessed and compared. Survival data were available for 121 patients in the GBM BVZ subgroup with a mean survival time of 295.9 days and for 277 patients in the BVZ control subgroup with a mean survival time of 355.1 days. Using these survival data and the Kaplan–Meier method, we compared the survival terms of these two subgroups, as shown in Fig. 3A. We found that patients with the GBM BVZ subtype had significantly shorter survival times than control patients (*p* = 0.014, case = 398). The average survival time between these two subgroups was shortened by approximately 60 days, indicating that the GBM BVZ subtype is more aggressive than the GBM control subtype.

In addition, the survival times of the other groups were also compared. Compared with the other three groups of DD, UD, and DU groups, there was only one significant difference (*p* = 0.023) between the GBM BVZ (UU) and DD subgroups; that is, both miR-21 and miR-10b were downregulated. Previously, we found that miR-21 was upregulated in many cancers^31^ and correlated with survival time in glioma patients^32^, so we compared all patients with miR-21 upregulation (UU + UD) to all patients with downregulated miR-21 (DU + DD). There was no significant change, possibly because all patients in this study were GBM patients, excluding patients with low-grade gliomas. Furthermore, GBM patients with only upregulated miR-10b did not affect their survival time.

We also compared the survival time of GBM patients associated with MGMT promoter methylation in these two subtypes (Fig. 3B and C). It was reported that tumors carrying MGMT promoter methylation have a more favorable prognosis for GBM patients^7^. However, these two GBM BVZ subtypes showed distinct survival patterns from MGMT promoter methylation. In the GBM control subtype but not the BVZ subtype, a significantly longer survival time association with MGMT promoter methylation was observed (*p* = 0.043).

We specifically assessed the gene methylation of VEGF, VEGFB, and VEGFC since we studied the BVZ response in GBM patients. Overall, VEGF methylation was much lower than that of VEGFB and VEGFC. However, there was a significant decrease in VEGF methylation in the GBM BVZ subtype compared to the control subtype (*p* = 0.005, case = 284), and there were no significant differences in VEGFB and VEGFC, as shown in Fig. 3D. This may contribute to differences in survival and BVZ response between these two GBM subtypes.

### Functional analysis using DE miRNAs

The functional characteristics of those DE miRNAs between these GBM BVZ subtypes were examined. Functional profiles of global co-DE miRNAs (i.e., significantly up- and downregulated miRNAs across all GBM samples between these two GBM subtypes) were examined using Gene Ontology (GO) enrichment analysis. As expected, these 14 DE miRNAs were involved in mRNA binding and gene silencing (molecular function (MF)), regulation of cytokine production (biological process (BP)), regulation of blood vessel endothelial cell proliferation in sprouting angiogenesis (BP), and more. More details are shown in Supplementary Table S1. However, when dividing these 14 DE miRNAs into globally upregulated and downregulated groups, researchers often do this^30^, and we found that these eight upregulated miRNAs are annotated in almost all functions that existed in the global analysis. Among the six downregulated miRNAs, one related to GO:1905001 had a different function than before, namely negative regulation of membrane repolarization during atrial cardiac muscle cell action potential (BP).

### Constructing three machine learning algorithms to classify and predict GBM BVZ subtypes based on miRNA z-scores

**Figure 4:**
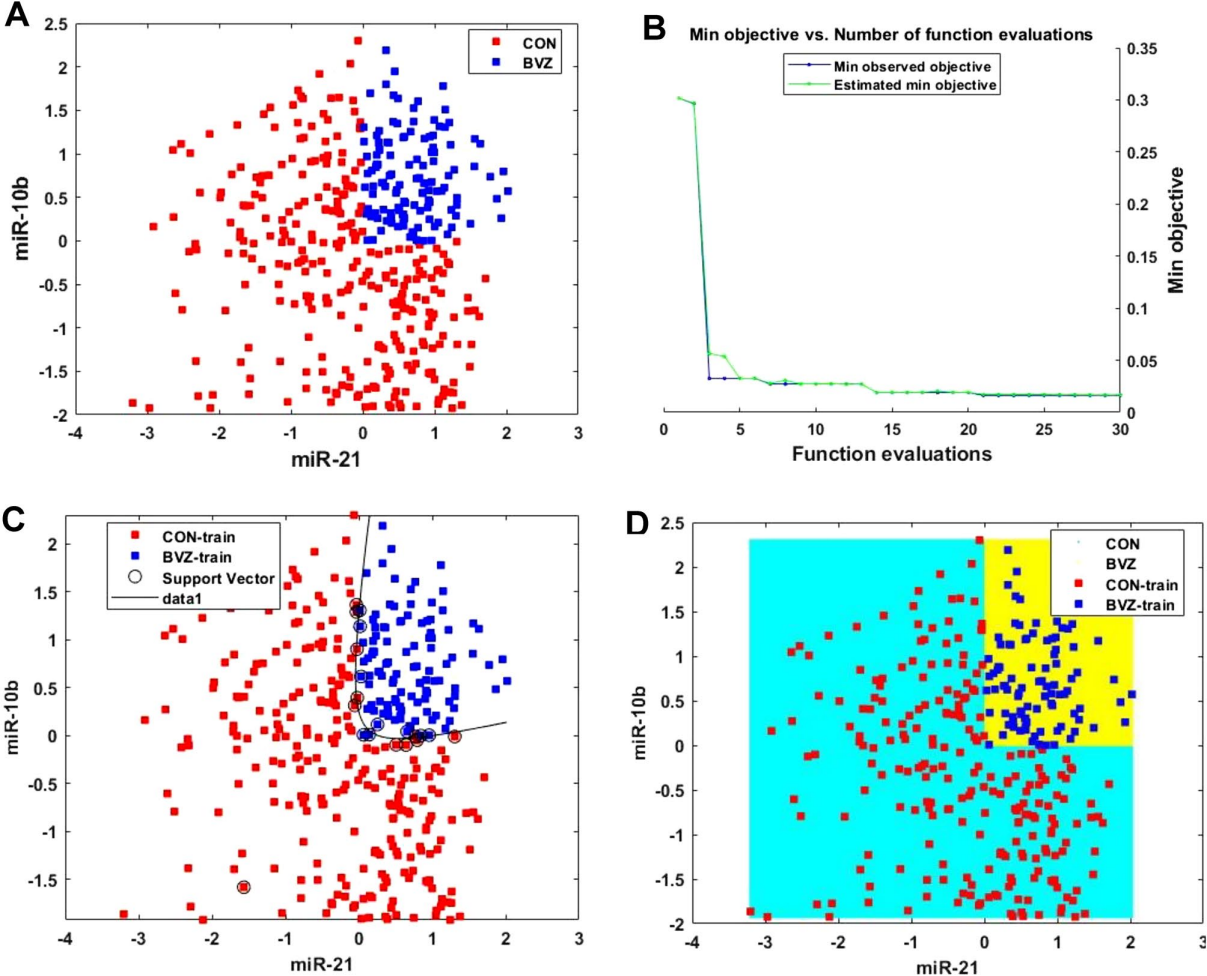
Constructing and comparing some classifiers for GBM BVZ subtypes based on miRNA z-scores of profiling. (**A**) For the z-score data points of the two subgroups, the horizon and vertical axes represent miR-21 and miR-10b. The blue or red dots are the GBM BVZ subgroup or the control subgroup. (**B**) The min. objective vs number of function evaluations during the construction of the SVM classifier. (**C**) The SVM model classifies GBM BVZ subtypes. All dots are the same as above, and the circles are the support vectors used. The curve is the edge of the two subgroups, and the accuracy is 100%. (**D**) The RF algorithm classifies GBM BVZ subtypes. The dots are in the same labels. If a blue or a red dot is in the yellow or cyan region, the prediction is correct; otherwise, it is wrong.

Based on the defined and classified GBM BVZ subtypes and the obtained matrices, we implemented a supervised machine learning classifier, the support vector machine (SVM) to train and test our previously defined datasets. In this study, we used the *fitcsvm*^33^ program and radial basis function (RBF) as its kernel function. The expression z-scores of miR-21 and miR-10b and the labels of those two subgroups were used in this step, as shown in Fig. 4A. Using a random number generator, the dataset was divided into an 80% training set and a 20% testing set. Given a set of training examples, each labeled as belonging to one of two categories, the SVM training algorithm built a model that assigned new examples to one category or the other. To build the model, the hyperparameters were automatically optimized by using the *fitcsvm*, in which the cross-validation loss was minimized.

The optimization processes of the SVM model are shown in Fig. 4B, in which the number of function evaluations is the iteration number of the objective function, and the min objective is the minimum value of the objective function reached in that iteration. The built model reached 100% accuracy on the test set, as shown in Fig. 4C. In this figure, those circles represent the support vectors used, the dots represent the training data, and the curve completely separates the two subgroups.

To compare different machine learning algorithms, the RF classifier was constructed by using the *fitctree*^33^ function and the same dataset. The results are shown in Fig. 4D, and its accuracy also reached 100%. In this picture, the blue or red dots represent the BVZ or CON training data, and if a blue or red dot is within the yellow or cyan region, the prediction is correct; otherwise, the prediction is wrong. In addition, an NN classifier was constructed by using the *fitcnet*^33^ program, optimizing to contain 15 first-layer nodes and 10 s-layer nodes, which also achieved 100% accuracy on the z-score dataset.

However, all these perfect classifications were based on the expression z-scores of miR-21 and miR-10b, which were represented by a batch of miRNA expression datasets^22^ and were difficult to use clinically.

### Constructing the SVM classifier to classify and detect GBM BVZ subtypes based on clinical datasets

To diagnose these GBM BVZ subtypes clinically, we have to develop a detection method for the subtypes based on current clinical techniques without the use of z-scores. In a previous study, to classify GBM BVZ subtypes, we used many datasets and controls. However, for the clinical tests so far, we typically use quantitative real-time PCR (qPCR) methods to diagnose patient samples without control samples. The qPCR analysis for testing miRNA expression is based on the use of endogenous controls for standardization, reliability, and reproducibility of results. For this purpose, we have to choose a control miRNA. After evaluating several candidates^34,35^, miR-16 was selected for two reasons: first, the expression of miR-16 was relatively high in all studied samples, and second, its expression level was stable in GBM subgroups, and its SD was relatively low compared to the target miRNAs.

After selecting miR-16 as the endogenous control, the microarray expression data selected in the above analysis were calculated to the log_2_-transformed ratios, which were performed as follows:

1 $$Ri \, = \, \log_{2} \left[ {\left( {miR - i} \right)/\left( {miR - 16} \right)} \right]$$

where Ri is the transformed ratio of control miR-16, and miR-i is one of the miRNA expression values. According to this formula, the expression values of miR-21 and miR-10b were divided by miR-16, and then the log_2_ of the ratio was calculated. There were some overlaps in this dataset compared to the same dataset of z-scores. After using this data set, the same methods of SVM (*fitcsvm*), RF (*fitctree*) and NN (*fitcnet*) were used as above, so they were still supervised machine learning problems. The SVM classifier achieved 90.2% accuracy rate, while the RF classifier achieved an 82.9% accuracy rate, the results of which are shown in Supplementary Fig. S1A and B. Furthermore, the NN classifier achieved an accuracy of 83.9%. Therefore, the SVM classifier was selected in the following research. Although this SVM classifier can be used for prediction, better accuracy can be achieved by optimizing and adding some variants.

To better predict the subtypes, we would like to add and try more variants. For this purpose, the *sequentialfs* function^33^ was used, and the variants were automatically optimized and selected among the 14 miRNAs obtained above. The optimization of feature subsets is that sequentialfs(fun, X, y) selects a subset of features from the data matrix X and sequentially compares features until the best candidate is found to best predict the data in y. In this study, the rows of the X matrix corresponded to patient observations; the columns of X corresponded to the 14 miRNAs obtained. In the first step, the *sequentialfs* selected each of the 14 miRNAs, kept calling the function and compared the accuracy until the best miR-21 was found. In the second step, the *sequentialfs* picked out each of the remaining 13 miRNAs to add miR-21 and consisted of each of the two miRNA panels, and then performed the same as before until the best two miRNA panel of miR-21 and miR-10b was found. Step-by-step evaluation of each possibility and each combination, including a panel of the 14 miRNAs, the best combination obtained from this process was the panel of miR-21, miR-10b, and miR-197. According to formula (1), the dataset for these three miRNA panel was calculated, and a new machine learning process of the SVM classifier was performed. This time, the prediction was indeed better than before, with the best accuracy rate reaching 95.1%. The overfitting problem of the SVM classifier was assessed by a cross-validation method before being used on other clinical datasets.

### K-fold cross validation for the SVM classifier

To prevent overfitting^36,37^, we performed stratified k-fold cross-validation for the SVM classifier since the distribution of the two subtypes was not equal. In this process, the *crossvalind*^33^ and *fitcsvm* programs were used. After fivefold cross validation, the average accuracy reached 85.4% (± 2.75, n = 5), suggesting no overfitting problem. In fact, most people think that SVM classifiers with rbf as its kernel function have difficulty producing the overfitting problem^38^.

After the cross validation, the confusion matrix, sensitivity, specificity, and mean accuracy of 85.4% were obtained, on which the receiver operating curve (ROC) of the classifier was generated, as shown in Supplementary Fig. S2A, and the confusion matrix is shown in Supplementary Fig. S2B. This classifier was then used for validation.

### Validation of the SVM classifier by using clinical datasets

To use this classifier widely, we tested several kinds of datasets, including different miRNA microarrays and real-time qPCR data. First, we used the miRNA expression dataset of 20 treated patients from the same dataset used for classifier construction, since there were no significant changes in miRNA expression compared to untreated miR-21, miR-10b, miR-197, and miR-16. After using the classifier and the dataset directly, we found that six patients belonged to the GBM BVZ subtype with a percentage of 30%, which is similar to the defined percentage we showed above. This validation seems to be very succsseful.

For the second validation, another miRNA expression profile (GSE25631) for malignant glioma tissues^39^ was used, in which GBM patients were Chinese, while the patients in the TCGA dataset used were mostly Caucasian patients. Although the microarray chips and detection methods were from the same company, the expression patterns of miRNAs were different. For example, the expression pattern of miR-197 was opposite to the microarray we used above (as shown in Supplementary Fig. S3), suggesting that miRNA expression may be different in different races. For this validation, we divided 81 GBM datasets (excluding one outlier) into 71 modification datasets and 10 validation datasets. The classifier was trained and modified using the dataset of 71 GBM samples until it reached 92.3% accuracy. After that, the classifier was used to detect the 10 remaining sample datasets, and the results were perfect for 3 BVZ patients and 7 control patients with GBM. If the experimental methods and patients’ race are the same as the dataset used, this classifier can be directly used to detect GBM BVZ subtypes.

**Figure 5:**
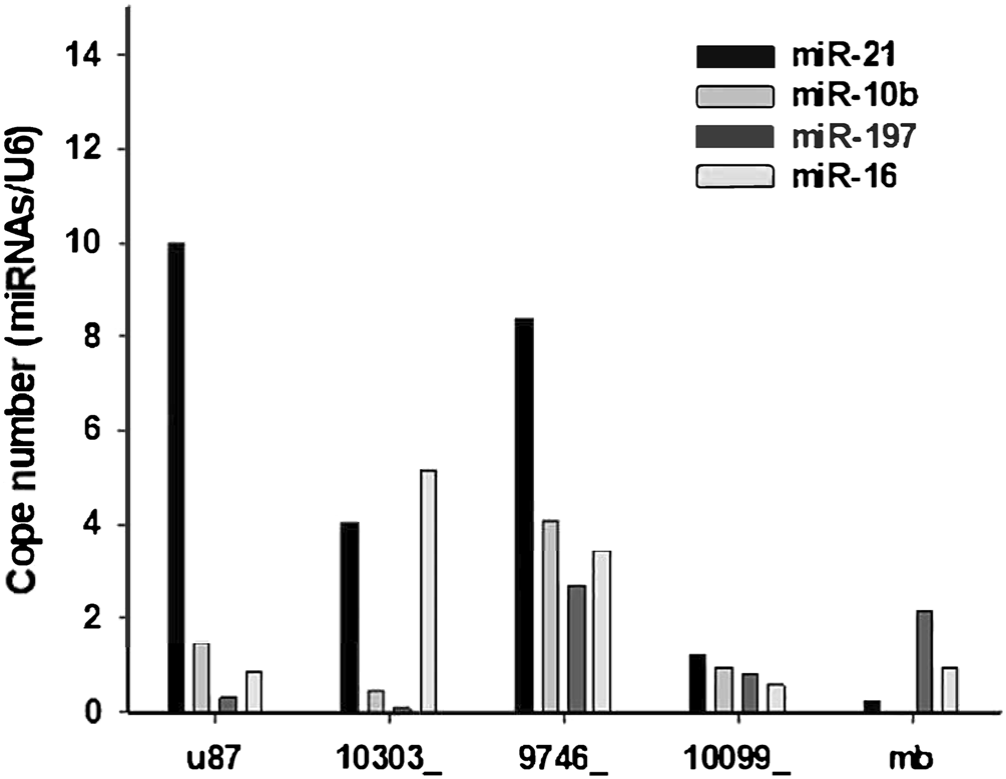
Relative miRNA expression in GBM tissues and cancer cell lines. In this study, the relative expression levels of four miRNAs, miR-21, miR-10b, miR-197, and miR-16, were used to detect GBM BVZ subtypes. The relative expression of miRNAs is expressed as the relative copy numbers of U6. In this picture, 10303_, 9746_, and 10099_ are GBM brain tissue samples, and U87 and mb are a GBM cell line and an MB cell line, respectively.

For the third validation, we performed a real-time qPCR experiment to obtain each Ct value of miR-21, miR-10b, miR-197, miR-16 and U6 from three GBM tissues, one GBM cell line, and one child brain tumor cell line. We found that the copy numbers of miRNAs can be used for this evaluation. To obtain the copy numbers for each miRNA, the standard curves of miR-21 and U6^40^ were used, and the average standard curve was calculated and used based on the standard curves of miR-31, miR-96, and miR-135b from the same study. According to the average standard curve, the copy numbers of miR-16, miR-10b, and miR-197 were obtained. According to the normalized equation: En = Copy number (target)/Copy number (reference)^41^, the relative expression levels of target miRNAs were obtained, as shown in Fig. 5. According to the formula (1), we prepared the qPCR dataset for this validation using the SVM classifier directly. One BVZ sample (10099) was found in those three GBM samples, and the positive rate was 33%. If U87 was counted, the rate was 25%. This is also a successful validation since the number of people can only be an integer and cannot be any decimal of a number.

## Discussion

Based on existing miRNA and mRNA profiling in this study, we classified and predicted GBM BVZ- responsive subtypes. Compared with other GBM patients, patients with the GBM BVZ subtype had a significantly shorter survival time. After using multiple bioinformatics methods between these subtypes, DE miRNAs and mRNAs were discovered, and their related functions and clinical characteristics were analyzed. Finally, the SVM classifier and the miRNA panel of miR-21, miR-10b, and miR-197 were provided for future clinical use, and they were examined and validated with several datasets. This study can be modified to classify and detect GBM clinical samples, and it may propose precise diagnosis and treatment ideas for GBM. Additionally, further clinical and basic research in GBM and other cancer studies is recommended.

By combining machine learning methods with the miRNA panel in this pilot study, GBM BVZ subtypes could be classified and predicted by existing miRNA profiling datasets, and they may also be detected and predicted from GBM clinical data. Previously, several GBM subclasses were classified by multiple miRNA and mRNA expression profiling^7,22^, but without artificial intelligence techniques, it is difficult to detect the classified GBM subclasses because multiple miRNAs cannot use some simple cutoff values of multiple biomarkers to separate different subclasses. As shown in Fig. 2B, there are four different subgroups for the expression of two miRNAs, and the actual edge between these two BVZ subtypes is a curve, not straight lines, as shown in Fig. 4C. However, we just wanted to make a small step progress in the precise diagnosis of GBM subtypes but met a big challenge. After multiple validations, we realized that presice diagnosis and treatment are far more complicated than traditional diagnosis and treatment. We discovered some problems in advance, including experimental techniques, patient ethnicity, data scales and ranges, which may affect the detection accuracy. Therefore, the classifier must be modified and trained with the same dataset before use. The pretraining dataset used cannot be too small. For example, when using a dataset with 37 GBM samples (GSE19870) obtained by different microarray platforms, we did not obtain a stable high-accuracy classifier.

In addition, since high levels of miR-10b and miR-21 were observed in the serum of some glioblastoma patients before treatment and during BVZ treatment compared with healthy controls and other patients^29^, this GBM BVZ subtype classification can be used not only to design therapeutic plans, but also to monitor treatment effects. Importantly, high expression levels of miR-10b and miR-21 were observed in the BVZ subtype of GBM. According to the five subclasses classified by multiple miRNAs^7^, patients with high expression of miR-21 and miR-10b were in different neural precursors or subgroups: astrocytic or oligoneural glioblastoma. These two glioblastoma subclasses have very different clinical and genetic distinctions. In addition, the combination of the panel of miR-21, miR-10b, and miR-197 with the SVM classifier for classification and detection of GBM BVZ subtypes has not been previously reported. Therefore, this BVZ subtype is a new subtype of GBM. In addition, since the survival time of patients with the GBM BVZ subtype was significantly shorter than that of other patients, people need to consider this when evaluating the therapeutic effects of BVZ.

In this study, 14 DE miRNAs were obtained between GBM BVZ subtypes, of which 7 have never been reported. This could be very important for future research. We found that miR-148a and miR-92a have been reported by our previous GBM study using the relative regression method^27^. In another study, miR-92 was also reported^42^. Of course, the presence of miR-21 and miR-10b among these miRNAs was the beginning of this study, and it is also a feedback support for the results obtained. As a tumor suppressor, downregulated miR-197 targeted GAB2 (Grb2-associated binding protein), releasing the inhibited proliferation in tumor cells^43^. Furthermore, in high-grade human gliomas, such as GBM, miR-197 was targeted and downregulated by downregulated FUS1 (TUSC2, tumor suppressor candidate 2), and downregulated miR-197 acted as a tumor suppressor to increase tumor cell proliferation^44^. Although Cai et al. reported that miR-542-3p was downregulated in glioblastoma cell lines, the expression level of miR-542-3p negatively correlated with the invasion of glioblastoma cells by targeting AKT1^45^, and different expression levels of miR-542 were present between GBM BVZ subtypes, as shown in Fig. 2C. Likewise, Pang et al. found that downregulation of miR-590-3p in GBM tissues and cell lines resulted in increased migration and invasion by targeting ZEB1/2 (zinc finger E-box 1/2)^46^, but miR-590 expression was reversed between the BVZ subtype and the control of GBM in this study. Importantly, those of unreported miRNAs and those reported miRNAs with unreported inverse expression patterns indicated very special miRNA expression patterns associated with these two GBM BVZ subtypes. This may be because other researchers used normal tissues as controls, whereas we used other GBM patients as controls rather than GBM patients with the BVZ subtype. Thus, these miRNAs are specifically associated with these GBM BVZ subtypes. For example, as shown in Fig. 2C, miR-197 expression was downregulated in the GBM BVZ subtype, but it was upregulated in its control subgroup, so it was selected as one of the variants for the SVM classifier after comparing all other combinations by the *sequencesf* program. In addition, miR-16 expression increased in GBM patients compared with normal controls^27,47^ and was maintained at the same expression level in GBM BVZ subtypes, so we chose it as the endogenous control.

Given the different functional profiles based on the 14 DE miRNAs obtained, we found that in addition to the common functions of miRNAs, some related functions are associated with VEGF. For general functions, RNA binding and gene silencing are routine functions of miRNAs, as shown in Supplementary Table S1. After analyzing the GO functional terms of all 14 DE miRNAs, several GO terms were clearly associated with VEGF, including regulation of blood vessel endothelial cell proliferation in sprouting angiogenesis (GO:1903587), blood vessel endothelial cell proliferation involved in sprouting angiogenesis (GO:0002043), and aortic smooth muscle cell differentiation (GO:0035887). These functions are very specific to VEGF, which is the design principle of the GBM BVZ subtypes and the feedback support for the results. Furthermore, if only 8 of the 14 miRNAs with increased expression were searched, more functions were associated with VEGF than previous functions, including regulation of endothelial cell proliferation (GO:0001936), endothelial cell proliferation (GO:0001935), and sprouting angiogenesis (GO:0002040). The reasons for the increased VEGF-related functions are unclear, suggesting that some functions may be hedged by negatively expressed miRNAs. However, if only negatively expressed miRNAs were analyzed, they had fewer relevant functions, suggesting that increased expression of miRNAs has dominant functions as inhibitors of mRNA expression, but the converse may not be the general case.

Therefore, to the best of our knowledge, this study is the first to analyze the mRNA and miRNA expression patterns associated with bevacizumab-responsive glioblastoma subtypes, indicating the existence of new GBM subtypes with clinical characteristics, DE miRNAs and mRNAs, and related specific functions. Importantly, we combined the SVM classifier and the panel of miRNAs to not only classify but also detect GBM BVZ subtypes for future clinical use. In addition, the study suggests that miR-21, miR-10b, and miR-197 can be used as potential GBM BVZ subtype biomarkers or monitors to help make therapeutic decisions and monitor treatment. This study may provide precise strategies for GBM treatment and future research.

## Supplementary Information

Supplementary Information.

## Data Availability

The raw datasets analyzed during the current study are available in The Cancer Genome Atlas (TCGA) repository [http://cancergenome.nih.gov/dataportal/].
