# Supplementary material for: Machine learning and bioinformatics approaches for classification and clinical detection of bevacizumab responsive glioblastoma subtypes based on miRNA expression
Source: Sci Rep. 2022 May 23;12:8685. doi: 10.1038/s41598-022-12566-x (PMC9126877; doi:10.1038/s41598-022-12566-x)
Supplement: Supplementary file 1 — Supplementary Information. [file 41598_2022_12566_MOESM1_ESM.pdf]

Supplementary Information

Source	Term Name	-LOG10(p)
GO:MF	RNA binding involved in posttranscriptional gene silencing	7.044533686
GO:MF	mRNA binding involved in posttranscriptional gene silencing	7.044533686
GO:MF	mRNA binding	5.379949536
GO:MF	mRNA 3'-UTR binding	4.999431533
GO:MF	RNA binding	2.636641753
GO:BP	gene silencing by miRNA	6.866746351
GO:BP	post-transcriptional gene silencing by RNA	6.816330049
GO:BP	posttranscriptional gene silencing	6.791483042
GO:BP	gene silencing by RNA	6.647180023
GO:BP	gene silencing	6.187240965
GO:BP	posttranscriptional regulation of gene expression	4.741993024
GO:BP	negative regulation of gene expression	3.540780231
GO:BP	regulation of blood vessel endothelial cell proliferation in sprouting angiogenesis	3.319663972
GO:BP	aortic smooth muscle cell differentiation	3.252517619
GO:BP	regulation of aortic smooth muscle cell differentiation	3.252517619
GO:BP	blood vessel endothelial cell proliferation involved in sprouting angiogenesis	3.155460339
GO:BP	miRNA mediated inhibition of translation	2.419265504
GO:BP	negative regulation of translation, ncRNA-mediated	2.419265504
GO:BP	regulation of translation, ncRNA-mediated	2.406850992
GO:CC	extracellular space	2.136945023

Table S1. Functional characteristics based on the differntial expressed 14 miRNAs.
The table shows all significant regulatory pathways between GBM BVZ subtypes based on the obtained 14 DE miRNAs by using the g:Profier tool and setting the threshold at p=0.01.

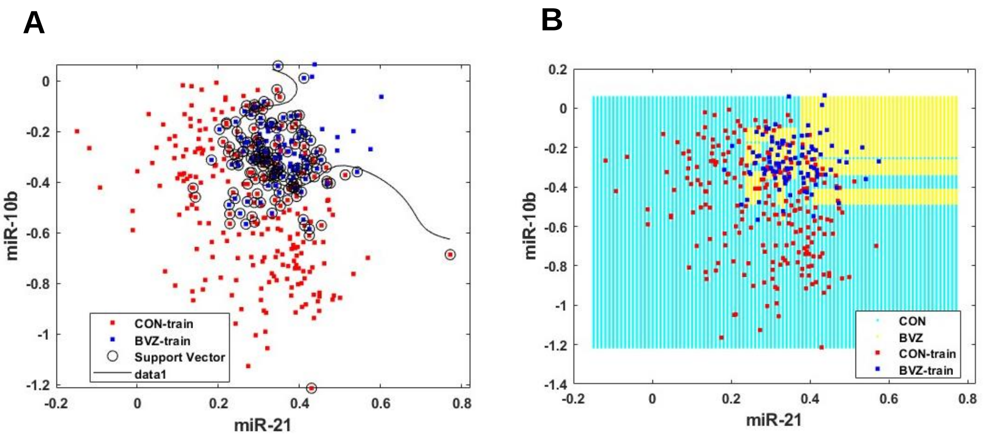

Fig. S1. Construction of SVM and RF classifiers for GBM BVZ subtypes based on clinical data

A) The SVM model classified GBM BVZ subtypes. The blue or red dots represent the training data for the BVZ or CON subtypes. The circles are support vectors used. The curve is the edge of two groups, and the accuracy is 90.2%. B) The RF model classified GBM BVZ subtypes with 82.9% accuracy. The blue or red dots represent the same thing as above. If a blue dot is in the yellow area, the prediction of the GBM BVZ subtype is correct; whereas if a red dot is in the cyan region, the prediction of the GBM CON subtype is correct. Otherwise, the prediction is wrong.

A

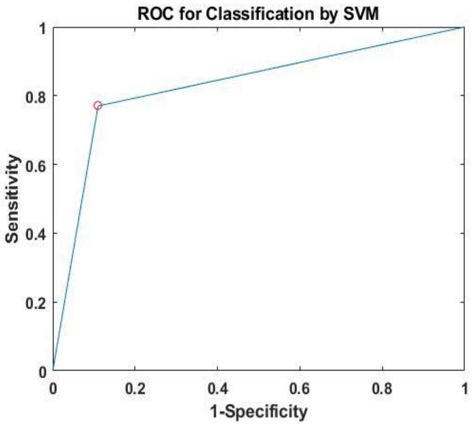

B

N=409		Actual Values	
		Positive	Negative
Predicted Values	Positive	(TP) **95**	(FP) **28**
	Negative	(FN) **32**	(TN) **254**

Figure S2. The receive operating curve (ROC) and the confusion matrix for the SVM classifier cross-validation. A) After 5-fold cross validation for the SVM classifier, the ROC was obtained, in which the red dot represented the average accuracy of 85.4%, sensitivity of 77.2%, and specificity of 88.8 %. **B)** The confusion matrix is shown for this cross-validation of the SVM classifier.

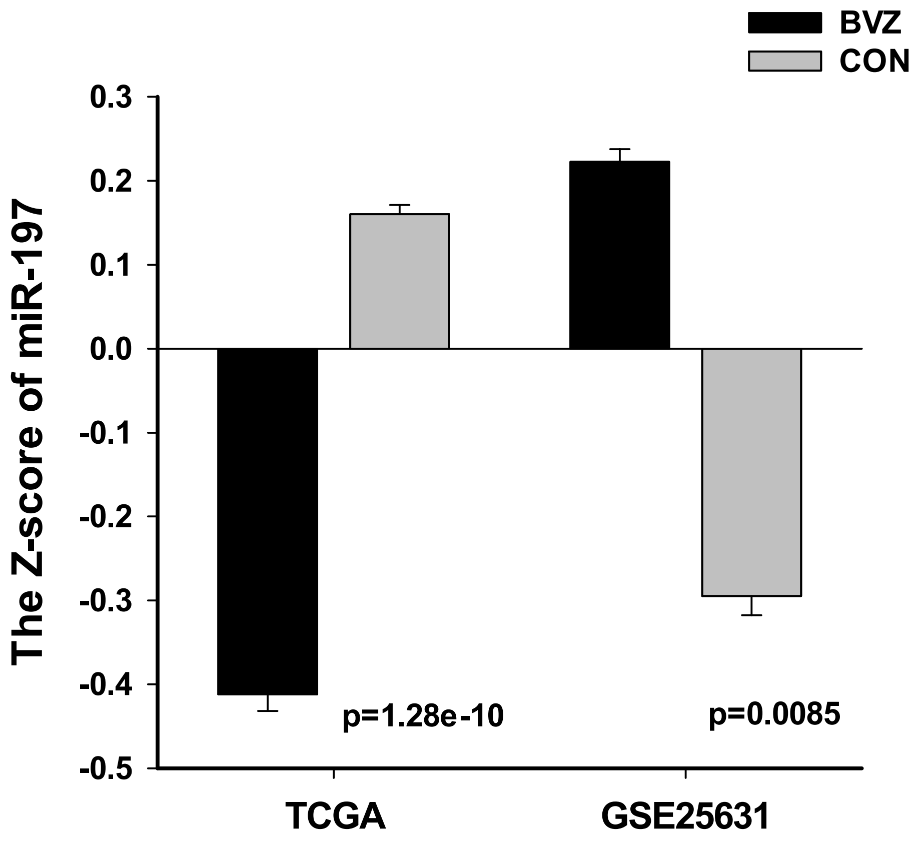

Figure S3. The miR-197 expression z-scores in TCGA and GSE25631 datasets used. In TCGA dataset used, the miR-197 z-score is negative in the BVZ subtype and positive in the control subtype of GBM. In contrast, the miR-197 z-score is positive in the BVZ subtype and negative in the control subtype of GBM in the GSE25631 GBM dataset.
